# Antioxidant, antimicrobial, and cytotoxic activities of extracts from the seed and pulp of Jujube (*Ziziphus jujuba*) grown in Iran

**DOI:** 10.1002/fsn3.2031

**Published:** 2020-12-04

**Authors:** Ahmad Rajaei, Davoud Salarbashi, Najmeh Asrari, Bibi Sedigheh Fazly Bazzaz, Seyyed Mohammad Aboutorabzade, Rezvan Shaddel

**Affiliations:** ^1^ Department of Food Science and Technology Faculty of Agriculture Shahrood University of Technology Shahrood Iran; ^2^ Department of Food Science and Nutrition School of Medicine Gonabad University of Medical Sciences Gonabad Iran; ^3^ School of Pharmacy Mashhad University of Medical Sciences Mashhad Iran; ^4^ Biotechnology Research Center Pharmaceutical Technology Institute Mashhad University of Medical Sciences Mashhad Iran; ^5^ Pharmaceutical Control Department School of Pharmacy Mashhad University of Medical Sciences Mashhad Iran; ^6^ Department of Medicinal Chemistry School of Pharmacy Mashhad University of Medical Sciences Mashhad Iran; ^7^ Department of Food Science and Technology Faculty of Agriculture and Natural Resources University of Mohaghegh Ardabili Ardabil Iran

**Keywords:** antimicrobials, antioxidant activity, cytotoxicity, jujube

## Abstract

The aim of the present study was to investigate the biological activities of the ultrasound‐assisted extracts obtained from pulp and seed of jujube (*Ziziphus jujuba*) fruits. To reach this purpose, total phenolic content (TPC), total flavonoid content (TFC), total pro‐anthocyanin, DPPH radical scavenging activity, rancimat test, as well as antimicrobial activity and cytotoxicity test of both jujube pulp and seed extracts were evaluated. Total phenolic content (TPC), total flavonoid content (TFC), and total pro‐anthocyanin in pulp extract were higher than those obtained from seed extract. In addition, DPPH radical scavenging activity of pulp extract (IC_50_ = 53.97 µg/ml) was higher than that of seed extract (IC_50_ = 88.68 µg/ml). Furthermore, the highest antimicrobial activity was observed against *Escherichia coli* and *Staphylococcus aureus* (MIC = 20 mg/ml) for both seed and pulp extracts. In vitro cytotoxicity evaluation on seven cell lines revealed that pulp and seed extracts of jujube had no cytotoxic activity. The present results suggested the promising antioxidant properties of jujube, which can be used in the fabrication of functional bioactive ingredients for different purposes.

## Graphical abstract

Jujube cultivated in Iran is a rich source of phenolic compounds. The extracts from seed and pulp showed good antioxidant activity. They had no cytotoxicity on human cells.

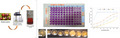

## 1 INTRODUCTION

Although oxidation is critical for plenty of organisms to produce energy for biological systems, oxygen‐derived free radicals (ODFR) from uncontrolled products can damage cells (Zhang et al., 2010). In recent years, natural antioxidants obtained from natural sources have attracted much attention due to being safer with less side effects in comparison with synthetic antioxidants (Anagnostopoulou et al., 2006; Karimkhani et al., 2016; Shakeri et al., 2018). Hence, various researches have been performed to evaluate the antioxidant capacity of natural compounds. It is extensively accepted that the antioxidant capacity of extracts obtained from plants is attributed to total phenolic content (TPC) and total flavonoid content (TFC) (Wilfred & Nicholson, 2006). It has been documented that phenolics have various biological activities like antioxidant (Tohma et al., 2017), antimicrobial (Oliveira et al., 2016) as well as anti‐inflammatory activities (Zhen et al., 2016). Furthermore, phenolic compounds play a determinant role in preventing cancer and heart diseases (Aguilera et al., 2016). According to the literature, the risk of cardiovascular diseases (Hertog et al., 1995) and distinct types of cancer (Yi et al., 2006) decreased with increasing consumption of vegetables and fruits.

*Ziziphus* species are part of Rhamnaceae family that are commonly utilized as traditional medicine in the treatment of different diseases such as diabetes, fever, gastrointestinal illnesses, obesity, urinary problems, skin infections, bronchitis, diarrhea, insomnia, and anemia (Pawlowska et al., 2009). *Ziziphus* species are widely grown in various regions, including south and west Asia, north India, south‐central China and also in southeastern Europe (Wojdyło et al., 2016). Environmental conditions have considerable effects on the amount of phenolic compounds in plants (Gao et al., 2011). Extraction is the primary step for obtaining the bioactive ingredients from various parts of plants. Maceration extraction (ME) as a prevalent method of extraction is very time‐consuming and needs relatively large volumes of solvents. Thus, novel techniques which need shorter extraction time and less organic solvent are of great interest. Ultrasound‐assisted extraction (UAE) as fast, more environmentally friendly and efficient method for chemicals extraction from solid plant matrixes would be a good alternative for conventional extraction (Heydari‐Majd et al., 2014; Wang & Weller, 2006). According to literature, jujube fruit and *Jatropha curcas* L. seeds that extract by organic solutions had stronger antioxidant activity than that obtained by DI water (Lin et al., 2020). The DPPH scavenging activity, total phenolic content, and reducing power of peel pulp of jujube are close to that of seed kernel of *Jatropha curcas* L. Accordingly, Singh et al (Singh et al., 2002) reported that the highest antioxidant yield was achieved with methanol compared to water and acetone.

In recent years, phenolic compounds of jujube cultivated in various regions such as China (Zhang et al., 2010), Korea (Choi et al., 2011), Italy (Pawlowska et al., 2009), India (Koley et al., 2016), Turkey (San & Yildirim, 2010), and Spain (Wojdyło et al., 2016) have been reported. However, there are no comprehensive data on the phenolic compounds and the antioxidant potential of jujube, grown under conditions of Iran. More information on the amount of phenolic compounds in medicinal plants such as jujube can provide an exceptional understanding of its antioxidant potential and increase consumption of this fruit to improve nutrition and increase the healthy food supply. This information is also useful for the process of jujube grown in the Iranian region for functional foods usage and as a combination with drugs and other nutrients.

Therefore, the present study was first aimed to evaluate total phenolic, flavonoid, and pro‐anthocyanin content of pulp and seed extracts from jujubes grown in Iran, then, to determine antioxidant capacity of pulp and seed extracts, and finally to evaluate antimicrobial activity and cytotoxicity of jujube pulp and seed extracts.

## 2 MATERIALS AND METHODS

### 2.1 Plant material and chemicals

The ripe jujube fruits, which selected by an expert based on fruits structure, flavor, and color, were first picked from South Khorasan Province (32.8653°N 59.2164°E), immediately transported to laboratory, and stored at 4°C. Afterward, jujubes with the uniform shape without any detectible diseases or blemishes were chosen. Then, the jujube fruits were cleaned with tap water and precisely separated into seed and pulp by a knife, and the pulp and seed of jujube fruits were dried in the shade. The obtained samples were powdered with a laboratory mill and dried powders passed through a standard 40 mesh sieve. Finally, the samples were stored at −18°C until further experiments. All the chemicals utilized in this study were provided from Merck.

### 2.2 Preparing methanolic extract

First of all, simple methanolic extracts of pulp and seed samples of jujube powered were obtained. To do this, five grams of pulp and seed samples of jujube powered were extracted with 100 ml of 80% (v/v) methanol during two 24‐hr phases. The mixture obtained upon one step extraction process were collected and stored in the refrigerator over filtering through Whatman No.1 filter paper. The dried and scraped plant residue remaining on the paper was weighed and was dissolved once again for 24 hr in methanol (1:20 w/v) and then was filtered. The subsequent procedures were mixing and concentrating of the filtrates obtained from the two extraction phases (4001 efficient, HeidolphLaborota) at 35◦C. Removing of traces of solvent was done using a vacuum oven (Labtech) at 35◦C (partial vacuum, 60 cm Hg) till reaching to the constant weight. Final samples were kept in desiccator until further analysis (Salarbashi et al., 2014).

### 2.3 Ultrasound‐assisted extraction

In second step, ultrasound‐assisted methanolic extracts of pulp and seed samples of jujube powered were obtained for comparison. High‐intensity ultrasound probe system of 24 kHz and 200 W (Dr.Hielscher, model UP 200H; GmbH) was applied to ultrasound‐assisted extraction with a 2 mm (S2) horn fitted of microtip immersed in a water bath in which a precipitate glass with the sample (1 g) was placed (internal dimensions: 280:195:135 mm). Intensity of ultrasonic vibrations and acoustic power was 21.8346 W/cm^2^ and 0.171402 W, respectively. The determination of the ultrasonic intensity was conducted calorimetrically via measuring the time–temperature increment of the solvent under adiabatic circumstances (Margulis & Margulis, 2003). The extractions were carried out at 35°C (ensured by a temperature controller coupled to the ultrasonic bath). A refrigerated centrifuge (Sigma, model 2–16 KC; Laborzentrigugen GmbH) was used to removing the insoluble part. After extraction, the residue of solvent was evaporated by rotary evaporator (HeidolphLaborota 4001 efficient). Drying of soluble part was done using a vacuum oven. All reported data in this manuscript are for ultrasound‐assisted extracts due to their high yield and quality as compared with simple methanolic extracts.

### 2.4 Total phenolic content (TFC)

Total phenolic contents in both seed and pulp extracts of jujube were evaluated using Folin–Ciocalteau technique with slight modifications (Heim et al., 2002). Briefly, 100 µl of the samples was first transferred to a glass tube and then were diluted with distilled water to 0.5 ml. Folin–Ciocalteau reagent (0.25 ml) and sodium carbonate (1.25 ml, 20% w/v) were then added to each tube, respectively. Afterward, the mixtures were stored for 40 min at 25 ºC, and finally, the absorbance of each sample was read at 725 nm by UV–vis spectrophotometer (Shimadzu UV‐VIS 1601). Control sample was distilled water. The TFC was reported as gallic acid equivalent (GAE) per g dried extract. All the experiments were performed in triplicate.

### 2.5 Total flavonoid content (TFC)

In order to determine TFC of the extracts, the colorimetric method was used as described by Muhamad et al (Muhamad et al., 2014). This technique is in accordance with the formation of flavonoid‐aluminum complexes with a maximum absorption at 410–430 nm. To reach this goal, 1 ml of the sample was first mixed with 1 ml of aluminum chloride (2% w/v) and then incubated at room temperature for 15 min. Finally, the absorbance of the mixture was recorded at 430 nm via spectrophotometer. TFC was expressed as mg of quinine (QEs) per g of dried extract. All the experiments were performed in triplicate.

### 2.6 Total pro‐anthocyanidins

To measure total pro‐anthocyanidins of the extracts, 1 ml of extracts was first added to 2 ml of vanillin solution (1% w/v). After 15 min, the absorbance of mixtures was read at 500 nm via spectrophotometer. The concentration of pro‐anthocyanidins was expressed as (+)‐catechin equivalents based on a standard curve for (+)‐catechin (Vermerris & Nicholson, 2007).

### 2.7 DPPH assessment

The antiradical potential of the pulp and seed extracts of Jujube was evaluated using DPPH assessment (Rajaei et al., 2010). Briefly, different concentrations of the extracts were first added to DPPH solution (1,000 µl, 0.012 g.100/ml), and kept for 2 hr in dark place. Then, the absorbance of the solutions was read at 517 nm via a spectrophotometer. Blank sample was pure methanol. BHT and vitamin C were employed as positive control. DPPH scavenging activity was determined as follows:

(1) $$\%\text{DPPH}=\frac{A_{b}‐A_{s}}{A_{b}}\times 100,$$

where, A_b_ is the control sample absorbance, and A_s_ is the samples absorbance. IC_50_ (extract concentration with 50% DPPH inhibition) was determined using plotting the inhibition (%) vs. the extract concentration(Habibi‐Nodeh et al., 2019).

### 2.8 Rancimat test

The oxidative stability index of fixed amount the investigated oil samples (4 g) containing Jujube extracts (300 mg/L including propylene glycol as emulsifier) was monitored at 120◦C by a Rancimat apparatus (Model 734) under 15 L/hr air flow. The volatile acids formed under accelerated conditions were trapped into the vessel including distilled water and conductometry of these acids was determined. Water conductivity measured over time (hr) and was calculated as µs/cm. The oxidative stability was expressed as the induction period (hr) of each oil sample corresponding to the time. Samples were analyzed simultaneously in triplicate (Delfanian et al., 2016; Habibi‐Nodeh et al., 2019; Salarbashi et al., 2014).

### 2.9 Antimicrobial activity

#### 2.9.1 Microorganism strains

Antimicrobial activity of the extracts against different bacteria including *Bacillus cereus* (PTCC 1247), and *Staphylococcus aureus* (Persian Type Culture Collection [PTCC] 1431) as Gram‐positive, as well as, *Salmonella typhi* [PTCC 1609] and *Escherichia coli* O157 [PTCC 1533] as Gram‐negative bacteria were examined. Microorganisms were supplied from IROST (Iranian Research Organization for Science and Technology).

#### 2.9.2 Agar dilution method

To evaluate the antimicrobial potential of seed and pulp extracts of Jujube, first, a fresh culture of every microorganism, which was adjusted to about 10^6^ CFU/ml with sterile normal saline (0.9%), was prepared. Afterward, the extracts were dissolved separately in Mueller‐Hinton broth (MHB). In the next step, 180 μl of every concentration containing 20 μl of bacterial suspension was transferred in the wells of a 96‐well cell culture plate. To show the sterility of media, a negative control prepared from MHB without any additives. Afterward, the plates were incubated for 18 hr at 37°C. Finally, to determine the growth of microorganisms, 20 µl of triazolium chloride solution (5 mg/ml) was added to the samples and then color changes were examined by colorimetric method. In this method, the first well without red color was considered as the minimum inhibitory concentration (MIC). Moreover, the assessment of the minimum bactericidal concentration (MBC) was conducted using reculturing the contents of wells without color change, on Mueller‐Hinton agar plates for 18 hr at 37ºC. All the therapeutic procedures were performed three times to approve the results (Shakeri et al., 2019).

#### 2.9.3 Antimold activity

The mycelial growth inhibition (MGI) of the jujube pulp and seed ultrasound‐assisted extracts against *Aspergillus niger* ATCC 16404, *Penicillium expansum* CCMI 625, and *Rhizopus stolonifer* UBA6 was determined as per our method described previously in (Salarbashi et al., 2016). Briefly, an appropriate amount of jujube pulp or seed ultrasound‐assisted extracts were dispersed in sodium caseinate solution (5% w/w) under sonication. Each sample was then added to Sabouraud Dextrose Agar (SDA) medium. For negative control, the sterilized agar medium without jujube extracts was used. Nystatin was also utilized as a reference drug. Subsequently, the inoculated Petri dishes were incubated at 25°C for seven days. At the end of this period, the mean diameters of the area of the fungal‐colony expansion in the respective tested and negative control dishes were monitored.

#### 2.9.4 Cytotoxicity test

The MTT test is commonly used to evaluate cell viability by converting yellow tetrazole, 3‐(4,5‐dimethylthiazol‐ 2‐yl)‐2,5‐diphenyltetrazolium bromide (MTT) to purple formazan in living cells. MTT test was used to determine cytotoxicity of the seed and pulp extracts of Jujube. For this purpose, several cell lines including MCF‐7, PC3, DU‐145, C26, HepG2, Hella, HTC, A2780, and PCL12 were used. All the tested cells seeded into 96‐well plates, and then the plates were incubated for 24 hr at 37°C. Then, the extracts were incorporated into plates and again incubated for 24 hr. In the next step, a medium containing MTT reagent was replaced with previous one and incubated at 37 ºC for 3 hr. After the media removal, it was washed by phosphate‐buffered saline (PBS) and then 0.2 ml of DMSO was added to dissolving the formazan crystals, the plates were shaken, and the absorbance was read at 550 nm. The results of cytotoxicity were calculated by CalcuSyn software (version 2; BIOSOFT) and reported as the concentration, which inhibited cell growth by 50% (IC_50_).

### 2.10 Statistical analysis

The analysis was performed by one‐way analysis of variance (ANOVA) with SPSS Statistics version 16.0.

## 3 RESULTS AND DISCUSSION

### 3.1 Total phenolic and flavonoid contents

Phenolic compounds are usually used in food industry as antioxidants (Cuvelier et al., 1992; Maillard et al., 1996). Total phenolic, flavonoid, and pro‐anthocyanidin contents of Jujube pulp and seed ultrasound‐assisted extracts are given in Table 1. TPC of Jujube pulp was higher than that of the Jujube seed. Moreover, the same results were observed for the TFC and total pro‐anthocyanidins (Table 1). Thus, from a nutritional point of view, the extract obtained from Jujube pulp is better than that of jujube seed. Also, Zhang et al. (2010) reported similar results that total phenolic and flavonoid contents of jujube pulp were higher than that of the Jujube seed.

**Table 1:** Total phenolic, flavonoids, and pro‐anthocyanins of jujube pulp and seed ultrasound‐assisted extracts

Sample	Total phenolic (mg TAE/g DW)	Total flavonoids (mg quercetin/g DW)	Pro‐anthocyanins (mg catechin/g DW)
Pulp	40.27 ± 0.15^a^	71.53 ± 0.36^a^	24.61 ± 0.03^a^
Seed	4.33 ± 0.01^b^	9.77 ± 0.01^b^	4.93 ± 0.01^b^

Note

There were no significant differences among the samples with the same letters in the same column (*p* < .05).

The moisture content of the fresh Jujube pulp was about 80%, and therefore TPC was 805.4 (mg GAE/100 g FW) based on the fresh weight (FW). Comparatively, TPC of jujube grown in Iran was higher than those reported for Chinese jujube (275–541 mg/100 g) (Q. H. Gao et al., 2012), Indian jujube (172–309 mg/100 g) (Hoshyar et al., 2015), and Spanish jujube (14.4–43.3 mg/g) (Wojdyło et al., 2016). This observation might be due to several reasons such as agronomic practices, genotype, harvesting time as well as postharvest and arid climatic conditions in Iran. Additionally, high‐altitude UV radiation can increase the accumulation of anthocyanins and other UV‐absorbing substances like total polyphenols and flavonoids in plants (Gao et al., 2011). Comparatively, the phenolic content of jujube pulp extract cultivated in Iran was higher than those reported for other relevant fruits known for their high phenolic content, such as pineapple (40.4 mg GAE/100 g FW), orange (56.8 mg GAE/100 g FW), peach (65.3 mg GAE/100 g FW), cranberry (507.0 mg GAE/100 g FW), apple (272.1 mg GAE/100 g FW), red grape (182.0 mg GAE/100 g FW), and strawberry (147.8 mg GAE/100 g FW (Delfanian et al., 2016). Thus, the jujube, especially those that have been cultivated in Iran, are rich in phenolics and could be introduced as a promising source of dietary phenolics. Furthermore, due to the considerable amounts of phenolic compounds, this natural source of antioxidant can be introduced as a suitable candidate for inhibition of lipid oxidation (Bisht et al., 2007).

Flavonoids are of considerable interest because of their protective effect against oxidative stress and ability to scavenge free radical (Xu & Chang, 2007). Moreover, based on the available literature (Rice‐Evans et al., 1996; Xu et al., 2010), flavonoids have diverse range of biological activities, for instance, they have been known as anti‐inflammatory, anticancer, antiallergic, and antioxidative agents (Mohtashami et al., 2019). As presented in Table 1, the TFCs and pro‐anthocyanidins of jujube pulp were significantly higher than those of seed extracts. Therefore, it is expected that antioxidant activity of jujube pulp extract can be higher than seed extracts, which will be investigated in the following section.

### 3.2 Antioxidant activity

**Figure 1:**
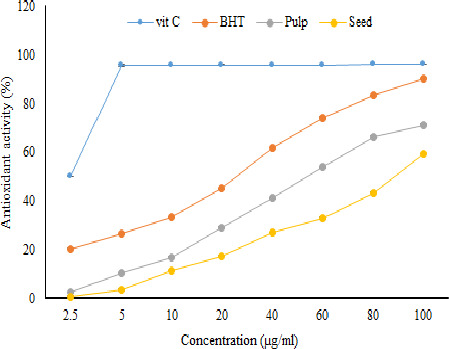
Antioxidant activity (%) of pulp and seed ultrasound‐assisted extracts of jujube determined by DPPH assay (*n* = 3)

The DPPH assay is extensively utilized to determine the antioxidant capacity of various plant extracts (Salehi et al., 2019). As shown in Figure 1, the antioxidant capacity of both seed and pulp extracts of jujube was concentration dependent. Figure 1 shows that the concentration dependency of the pulp extract was higher than that of the seed extract. The difference between the antioxidant capacity of the seed and pulp extracts of jujube may be due to the higher content of TPC, TFC, and pro‐anthocyanidins in the pulp extract compared to the seed extract which was confirmed in above section.

The IC_50_ values of pulp and seed ultrasound‐assisted extracts of jujube were higher than the IC_50_ of vitamin C and BHT, which may be due to the existence of impurities other than phenolic compounds in the extracts of jujube seed and pulp (Table 2). Moreover, the IC_50_ of pulp extract was lower than the IC_50_ of seed extract, which is presumably related to higher phenolics and flavonoids of pulp extract. It should be noted that less IC_50_ indicates higher antioxidant activity and vice versa. The antioxidant capacity of jujube cultivated in Iran was higher than two varieties of Korean jujube. Choi et al. (2012) reported that the IC_50_ values of seed extracts of two varieties of Mechu and Sanzoin jujube cultivated in Korea were 310.2 (µg/ml) and 195.4 (µg/ml), respectively. Furthermore, the IC_50_ values of pulp extracts of Mechu and Sanzoin were 115.6 (µg/ml) and 145.8 (µg/ml), respectively. Similarly, they have shown that the antioxidant capacity of jujube pulp extract was higher than that of the jujube seed extract. The greater amount of phenolic compounds in the jujube cultivated in Iran as well as the type of phenolic compounds can be the primary reasons for the better antioxidant capacity of our samples.

**Table 2:** DPPH radical scavenging activity (IC_50_) of jujube pulp and seed ultrasound‐assisted extracts (µg/ml)

Sample	IC_50_ (µg/ml)
Pulp	53.97
Seed	88.68
BHT	13.47
Vit. C	9.17

### 3.3 Oxidative Stability Index (OSI)

As proved before, prediction of the oxidative stability index by Rancimat is the most well‐known applied indicators to measure oxidative stability of edible fats and oils under simulated and accelerated conditions (Velasco et al., 2009). The oxidative stability of the jujube samples was evaluated by measuring their IPs with Rancimat method. OSI results were in agreement with results reported by Delfanian et al. (2016). The values of IP measured at 110°C showed that jujube pulp oil (3.70 hr) was more stable than jujube seed oil (3.65 hr) (*p* < .05).

TPC and TFC contents of oils are considered as one of the most significant parameter influencing the oxidation susceptibility of the oils (Tohidi et al., 2017). Higher amount of TPC and TFC in the pulp extract resulted to lower susceptibility. The effectiveness of the higher phenolic concentrations of the extracts in resistance of the oils has been confirmed (B. Xu & Chang, 2007; Zhang et al., 2010). Some authors reported that the IP values of the jujube fruits obtained using Rancimat method have been correlated positively to their antioxidant activity (Delfanian et al., 2016). A comparison between the results of the IP and IC of Jujube extracts suggested that the jujube pulp with greater IP values had a higher antioxidant activity. However, the seed and the pulp of the jujube fruit IP values were not significantly difference (*p* < .05).

### 3.4 Antimicrobial activity

The MIC and MBC of seed and pulp extracts of jujube for different bacterial strains are given in Table 3. It can be seen that the MIC and MBC were equal for the three strains of *S. aureus*, *E. coli,* and *B. cereus*, but in the case of *S. typhi*, the MBC was higher than the MIC. The MIC and MBC of seed and pulp extracts for *E. coli* were 20 mg/ml. According to this result, it can be concluded that *E. coli* was the most susceptible microorganism to the seed and pulp extracts among the tested microorganisms. On the other hand, the results exhibited that the compounds present in the extracts obtained from the seed and pulp of jujube had low antibacterial potential vs. *S. typhi as* a Gram‐negative bacteria. In addition, the MIC and MBC for *B. cereus* and *S. aureus* showed the similar sensitivity of both Gram‐positive strains to the extracts of jujube seed and pulp. The results of this study are important since *S. aureus* and *B. cereus* could produce different types of enterotoxins that cause gastroenteritis.

**Table 3:** Minimum inhibitory concentrations (MICs) and minimum bactericidal concentrations (MBCs) of *Zizyphus jujube* ultrasound‐assisted extracts

Bacterial Spp.	PTCC	Pulp extract (mg/ml)		Seed extract (mg/ml)	
		MBC	MIC	MBC	MIC
*S. aureus*	1,431	40	40	40	40
*E. coli O157*	1,533	20	20	20	20
*B. cereus*	1,247	40	40	40	40
*S. typhi*	1,609	160	20	20	80

The results of this work were in agreement with those reported by Ghazghazi et al. (2014), who evaluated the antibacterial potential of jujube extract on several Gram‐negative and Gram‐positive bacteria. They found that the MIC values for *S. typhi*, *E. coli*, *S. aureus,* and *B. cereus* were 25, 25, 50, and 50 (mg/ml), respectively. Previous studies reported that Gram‐positive bacteria are more sensitive to the extracts of plants than Gram‐negative bacteria owing to hydrophobic lipopolysaccharide in the outer membrane which provides more protection (Shakeri et al., 2014). Nevertheless, the achieved results showed that the extracts did not possess selective antibacterial capacity.

Many studies have been reported the antimicrobial potential of phenolic compounds (Bouarab‐Chibane et al., 2019). However, the mechanism of these compounds has not been clearly elucidated. Santiesteban‐López, Palou, and López‐Malo (2007), for instance, reported that the effect of phenolic compounds was concentration‐dependent, and at low concentrations, phenolic compounds affect the activity of enzymes, especially those related to energy production. The effect of phenolic compounds on microbial growth and toxin production may also be due to the ability of phenolic compounds to change the permeability of cell walls. In other words, these compounds can react with membrane proteins and consequently change their function. Stratford and Eklund (2003) exhibited that phenolic compounds can affect the physiological responses of microorganisms. Moreover, phenolic compounds can abnormalize the enzymes responsible for spore growth or interfere with the production of the amino acids required for the growth process. Furthermore, literature review demonstrated that different phenolic compounds had various effects on *S. aureus* membranes (Kwon et al., 2007; Miklasińska‐Majdanik et al., 2018). Therefore, phenolic compounds may not have a common mechanism and may have different targets in relation to their antimicrobial activity.

**Figure 2:**
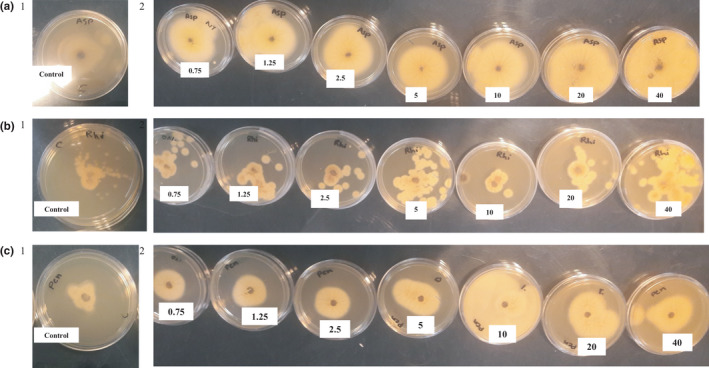
Image of antimold activity of Jujube seed/pulp extract (control [1] and Jujube seed/pulp extract at different concentrations [2])

According to the antimold study (Figure 2a‐c), jujube not only failed to inhibit the growth of the three studied molds (*Aspergilus niger*, *Penicillium expansum,* and *Rhizopus stolonifer*), but also strengthened their growth in higher concentrations which may be due to the high sugar content of jujube extracts.

### 3.5 Cytotoxicity

**Figure 3:**
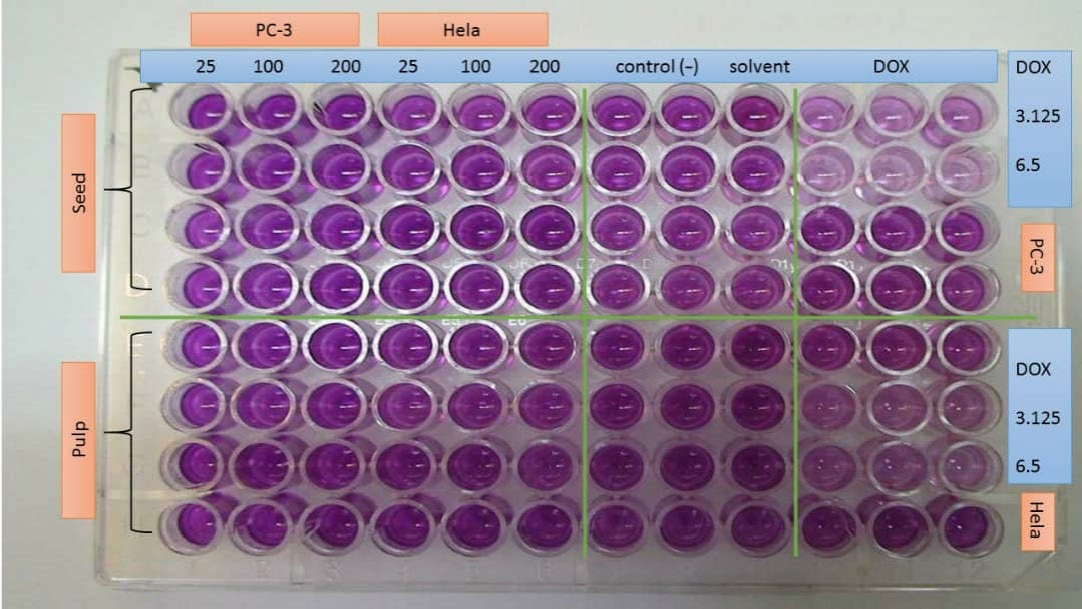
The photographs for cytotoxic activity in 96 well plate cell culture

The cytotoxic activity of seed and pulp extracts of jujube has been presented in Table 4. In addition, the photographs for cytotoxic activity in 96 well plate cell culture for the PC3 and Hella tested cancerous cells have been represented in Figure 3. As can be seen, two cancerous cell lines of PC3 and Hella have been tested on Jujube seed and pulp extracts at three concentrations of 25, 100, and 200 mg/L. Negative control that contains cell lines and culture media showed color change indicating the viability of cancer cell. Also, the results of solvent containing culture media showed color change confirming that pure culture media had no cancer agent. Positive control contains cell lines, culture media, and doxorubicin (DOX). Doxorubicin in two concentrations of 3.125 and 6.50 mg/L has been entered into the PC3 and Hella tested cancerous cells leading to cell death. According to Table 4 and Figure 3, MTT test indicated no cytotoxicity effect on the tested cancerous cells (MCF‐7, PC3, DU‐145, HepG2, C26, HTC, Hella, PCL12, and A2780). Similar results have been reported by Iranian authors (Hoshyar et al., 2015) from the aqueous extract of jujube fruit who found the IC_50_ values of 1,800, 1,000, and 500 μg/ml after 24, 48, and 72 hr, respectively, against the breast cancer cells. In another study by Taechakulwanijya et al (Taechakulwanijya et al., 2016), it was shown that different extracts of the jujube seeds including water, dichloromethane, ethyl acetate, and hexane had no significant cytotoxic effect on against both Jurkat and Vero cell lines. In contrast, some other authors have found cytotoxic activity of jujube fruit extracts toward several cancer cell lines, except for normal cell lines (Plastina et al., 2012). Some others even have demonstrated that the mechanism of action of cytotoxicity of jujube is through the induction of apoptosis (Vahedi et al., 2008). Taken together, according to our results, the pulp and seed extracts of jujube had low antimicrobial activity, and being inert against cancerous cell lines, which might indicate that these extracts do not have carcinogenicity potential.

**Table 4:** Cytotoxic activity of *Zizyphus jujube* ultrasound‐assisted extracts

Cell line	IC_50_ (µg/ml)	
	Pulp extract	Seed extract
MCF−7	>N.A^a^	N.A
PC3	N.A	N.A
DU−145	N.A	N.A
HepG2	N.A	N.A
C26	N.A	N.A
HTC	N.A	N.A
Hella	N.A	N.A
PCL12	N.A	N.A
A2780	N.A	N.A

^a^ Not active.

## 4 CONCLUSION

The results of present research pointed out that jujube cultivated in Iran are a rich source of phenolic compounds. Its phenolic content was relatively higher than that of previously published in literature. Jujube seed was also the source of phenolic compounds like pulp. However, the phenolic compounds of jujube seed were lower than that of pulp. Furthermore, the extracts from seed and pulp showed good antioxidant activity. The extracts of jujube seed and pulp were to some extent effective in preventing growth of Gram‐negative and Gram‐positive bacteria. It should be noted that the extracts of jujube seed and pulp had no cytotoxicity on human cells. All in all, obtained results indicated that jujube cultivated in Iran is a potential source of phenolic compounds for use in food and pharmaceutical industries.

## CONFLICT OF INTEREST

It is declared that there is no conflict of interest in publication of this work.

## ETHICAL APPROVAL

Our research did not contain any animal experiments and human subjects.

## References

[fsn32031-bib-0001] Aguilera, Y. , Martin‐Cabrejas, M. A. , & de Mejia, E. G. (2016). Phenolic compounds in fruits and beverages consumed as part of the mediterranean diet: Their role in prevention of chronic diseases. Phytochemistry Reviews, 15(3), 405–423. 10.1007/s11101-015-9443-z

[fsn32031-bib-0002] Anagnostopoulou, M. A. , Kefalas, P. , Papageorgiou, V. P. , Assimopoulou, A. N. , & Boskou, D. (2006). Radical scavenging activity of various extracts and fractions of sweet orange peel (*Citrus sinensis*). Food Chemistry, 94(1), 19–25. 10.1016/j.foodchem.2004.09.047

[fsn32031-bib-0003] Bisht, S. , Feldmann, G. , Soni, S. , Ravi, R. , Karikar, C. , Maitra, A. , & Maitra, A. (2007). Polymeric nanoparticle‐encapsulated curcumin ("nanocurcumin"): A novel strategy for human cancer therapy. Journal of Nanobiotechnology, 5(1), 3 10.1186/1477-3155-5-3 17439648 PMC1868037

[fsn32031-bib-0004] Bouarab‐Chibane, L. , Forquet, V. , Lantéri, P. , Clément, Y. , Léonard‐Akkari, L. , Oulahal, N. , Degraeve, P. , & Bordes, C. (2019). Antibacterial properties of polyphenols: Characterization and QSAR (quantitative structure–activity relationship) models. Frontiers in Microbiology, 10(829), 1–23. 10.3389/fmicb.2019.00829 31057527 PMC6482321

[fsn32031-bib-0005] Choi, S.‐H. , Ahn, J.‐B. , Kim, H.‐J. , Im, N.‐K. , Kozukue, N. , Levin, C. E. , & Friedman, M. (2012). Changes in free amino acid, protein, and flavonoid content in jujube (*Ziziphus jujube*) fruit during eight stages of growth and antioxidative and cancer cell inhibitory effects by extracts. Journal of Agricultural and Food Chemistry, 60(41), 10245–10255. 10.1021/jf302848u 23046062

[fsn32031-bib-0006] Choi, S.‐H. , Ahn, J.‐B. , Kozukue, N. , Levin, C. E. , & Friedman, M. (2011). Distribution of free amino acids, flavonoids, total phenolics, and antioxidative activities of jujube (*Ziziphus jujuba*) fruits and seeds harvested from plants grown in Korea. Journal of Agricultural and Food Chemistry, 59(12), 6594–6604. 21574660 10.1021/jf200371r

[fsn32031-bib-0007] Cuvelier, M.‐E. , Richard, H. , & Berset, C. (1992). Comparison of the antioxidative activity of some acid‐phenols: Structure‐activity relationship. Bioscience, Biotechnology, and Biochemistry, 56(2), 324–325. 10.1271/bbb.56.324

[fsn32031-bib-0008] Delfanian, M. , Esmaeilzadeh Kenari, R. , & Sahari, M. A. (2016). Utilization of Jujube fruit (*Ziziphus mauritiana* Lam.) extracts as natural antioxidants in stability of frying oil. International Journal of Food Properties, 19(4), 789–801.

[fsn32031-bib-0009] Gao, Q. H. , Wu, C. S. , Yu, J. G. , Wang, M. , Ma, Y. J. , & Li, C. L. (2012). Textural characteristic, antioxidant activity, sugar, organic acid, and phenolic profiles of 10 promising jujube (*Ziziphus jujuba* Mill.) selections. Journal of Food Science, 77(11), C1218–C1225. 23057538 10.1111/j.1750-3841.2012.02946.x

[fsn32031-bib-0010] Gao, Q.‐H. , Wu, P.‐T. , Liu, J.‐R. , Wu, C.‐S. , Parry, J. W. , & Wang, M. (2011). Physico‐chemical properties and antioxidant capacity of different jujube (*Ziziphus jujuba* Mill.) cultivars grown in loess plateau of China. Scientia Horticulturae, 130(1), 67–72. 10.1016/j.scienta.2011.06.005

[fsn32031-bib-0011] Ghazghazi, H. , Aouadhi, C. , Riahi, L. , Maaroufi, A. , & Hasnaoui, B. (2014). Fatty acids composition of Tunisian *Ziziphus lotus* L. (Desf.) fruits and variation in biological activities between leaf and fruit extracts. Natural Product Research, 28(14), 1106–1110. 24805194 10.1080/14786419.2014.913244

[fsn32031-bib-0012] Habibi‐Nodeh, F. , Farhoosh, R. , & Sharif, A. (2019). Frying stability time of olive oils estimated from the oxidative stability index. Journal of Food Measurement and Characterization, 13(3), 1831–1838. 10.1007/s11694-019-00101-y

[fsn32031-bib-0013] Heim, K. E. , Tagliaferro, A. R. , & Bobilya, D. J. (2002). Flavonoid antioxidants: Chemistry, metabolism and structure‐activity relationships. The Journal of Nutritional Biochemistry, 13(10), 572–584. 10.1016/S0955-2863(02)00208-5 12550068

[fsn32031-bib-0014] Hertog, M. G. , Kromhout, D. , Aravanis, C. , Blackburn, H. , Buzina, R. , Fidanza, F. , & Nedeljkovic, S. (1995). Flavonoid intake and long‐term risk of coronary heart disease and cancer in the seven countries study. Archives of Internal Medicine, 155(4), 381–386. 10.1001/archinte.1995.00430040053006 7848021

[fsn32031-bib-0015] Heydari‐Majd, M. , Rajaei, A. , Salarbashi, D. , Mortazavi, S. A. , & Bolourian, S. (2014). Optimization of ultrasonic‐assisted extraction of phenolic compounds from bovine pennyroyal (*Phlomidoschema parviflorum*) leaves using response surface methodology. Industrial Crops and Products, 57, 195–202. 10.1016/j.indcrop.2014.03.031

[fsn32031-bib-0016] Hoshyar, R. , Mohaghegh, Z. , Torabi, N. , & Abolghasemi, A. (2015). Antitumor activity of aqueous extract of *Ziziphus jujube* fruit in breast cancer: An *in vitro* and *in vivo* study. Asian Pacific Journal of Reproduction, 4(2), 116–122. 10.1016/S2305-0500(15)30007-5

[fsn32031-bib-0017] Karimkhani, M. M. , Shaddel, R. , Hadad‐Khodaparast, M. H. , Vazirian, M. , & Piri‐Gheshlaghi, S. (2016). Antioxidant and antibacterial activity of safflower (*Carthamus tinctorius* L.) extract from four different cultivars. Quality Assurance and Safety of Crops & Foods, 8(4), 565–574.

[fsn32031-bib-0018] Koley, T. K. , Kaur, C. , Nagal, S. , Walia, S. , Jaggi, S. , & Sarika, S. (2016). Antioxidant activity and phenolic content in genotypes of Indian jujube (*Zizyphus mauritiana* Lamk.). Arabian Journal of Chemistry, 9, S1044–S1052. 10.1016/j.arabjc.2011.11.005

[fsn32031-bib-0019] Kwon, Y. I. , Apostolidis, E. , Labbe, R. , & Shetty, K. (2007). Inhibition of Staphylococcus aureus by phenolic phytochemicals of selected clonal herbs species of Lamiaceae family and likely mode of action through proline oxidation. Food Biotechnology, 21, 71–89. 10.1080/08905430701191205

[fsn32031-bib-0020] Lin, Y. S. , Lin, W. S. , Tung, J. W. , Cheng, Y. C. , Chang, M. Y. , Chen, C. Y. , & Huang, S. L. (2020). Antioxidant capacities of jujube fruit seeds and peel pulp. Applied Sciences, 10(17), 6007 10.3390/app10176007

[fsn32031-bib-0021] Maillard, M.‐N. , Soum, M.‐H. , Boivin, P. , & Berset, C. (1996). Antioxidant activity of barley and malt: Relationship with phenolic content. LWT‐Food Science and Technology, 29(3), 238–244. 10.1006/fstl.1996.0035

[fsn32031-bib-0022] Margulis, M. , & Margulis, I. (2003). Calorimetric method for measurement of acoustic power absorbed in a volume of a liquid. Ultrasonics Sonochemistry, 10(6), 343–345. 12927610 10.1016/S1350-4177(03)00100-7

[fsn32031-bib-0023] Miklasińska‐Majdanik, M. , Kępa, M. , Wojtyczka, R. D. , Idzik, D. , & Wąsik, T. J. (2018). Phenolic compounds diminish antibiotic resistance of *Staphylococcus aureus* clinical strains. International Journal of Environmental Research and Public Health, 15(10), 2321 10.3390/ijerph15102321 PMC6211117 30360435

[fsn32031-bib-0024] Mohtashami, L. , Shakeri, A. , & Javadi, B. (2019). Neuroprotective natural products against experimental autoimmune encephalomyelitis: A review. Neurochemistry International, 129, 104516 10.1016/j.neuint.2019.104516 31376428

[fsn32031-bib-0025] Muhamad, N. , Muhmed, S. , Yusoff, M. , & Gimbun, J. (2014). Influence of solvent polarity and conditions on extraction of antioxidant, flavonoids and phenolic content from *Averrhoa bilimbi* . Journal of Food Science and Engineering, 4, 255–260. 10.17265/2159-5828/2014.05.006

[fsn32031-bib-0026] Oliveira, B. D’. Á. , Rodrigues, A. C. , Cardoso, B. M. I. , Ramos, A. L. C. C. , Bertoldi, M. C. , Taylor, J. G. , Cunha, L. R. D. , & Pinto, U. M. (2016). Antioxidant, antimicrobial and anti‐quorum sensing activities of *Rubus rosaefolius* phenolic extract. Industrial Crops and Products, 84, 59–66. 10.1016/j.indcrop.2016.01.037

[fsn32031-bib-0027] Pawlowska, A. M. , Camangi, F. , Bader, A. , & Braca, A. (2009). Flavonoids of *Zizyphus jujuba* L. and *Zizyphus spina‐christi* (L.) Willd (Rhamnaceae) fruits. Food Chemistry, 112(4), 858–862.

[fsn32031-bib-0028] Plastina, P. , Bonofiglio, D. , Vizza, D. , Fazio, A. , Rovito, D. , Giordano, C. , Barone, I. , Catalano, S. , & Gabriele, B. (2012). Identification of bioactive constituents of *Ziziphus jujube* fruit extracts exerting antiproliferative and apoptotic effects in human breast cancer cells. Journal of Ethnopharmacology, 140(2), 325–332. 10.1016/j.jep.2012.01.022 22301448

[fsn32031-bib-0029] Rajaei, A. , Barzegar, M. , Mobarez, A. M. , Sahari, M. A. , & Esfahani, Z. H. (2010). Antioxidant, anti‐microbial and antimutagenicity activities of pistachio (*Pistachia vera*) green hull extract. Food and Chemical Toxicology, 48(1), 107–112. 10.1016/j.fct.2009.09.023 19781589

[fsn32031-bib-0030] Rice‐Evans, C. A. , Miller, N. J. , & Paganga, G. (1996). Structure‐antioxidant activity relationships of flavonoids and phenolic acids. Free Radical Biology and Medicine, 20(7), 933–956. 10.1016/0891-5849(95)02227-9 8743980

[fsn32031-bib-0031] Salarbashi, D. , Asili, J. , Mohtashami, S. , & Farshchi, H. K. (2014). Composition of essential oil of *Oxytropis kuchanensis* from Iran. Chemistry of Natural Compounds, 50(1), 153–154. 10.1007/s10600-014-0897-9

[fsn32031-bib-0032] Salarbashi, D. , Mortazavi, S. A. , Noghabi, M. S. , Bazzaz, B. S. F. , Sedaghat, N. , Ramezani, M. , & Shahabi‐Ghahfarrokhi, I. (2016). Development of new active packaging film made from a soluble soybean polysaccharide incorporating ZnO nanoparticles. Carbohydrate Polymers, 140, 220–227. 10.1016/j.carbpol.2015.12.043 26876847

[fsn32031-bib-0033] Salehi, E. , Emam‐Djomeh, Z. , Askari, G. , & Fathi, M. (2019). *Opuntia ficus indica* fruit gum: Extraction, characterization, antioxidant activity and functional properties. Carbohydrate Polymers, 206, 565–572. 10.1016/j.carbpol.2018.11.035 30553358

[fsn32031-bib-0034] San, B. , & Yildirim, A. N. (2010). Phenolic, alpha‐tocopherol, beta‐carotene and fatty acid composition of four promising jujube (*Ziziphus jujuba* Miller) selections. Journal of Food Composition and Analysis, 23(7), 706–710. 10.1016/j.jfca.2010.02.008

[fsn32031-bib-0035] Santiesteban‐López, A. , Palou, E. , & López‐Malo, A. (2007). Susceptibility of food‐borne bacteria to binary combinations of antimicrobials at selected aw and pH. Journal of Applied Microbiology, 102(2), 486–497. 17241355 10.1111/j.1365-2672.2006.03092.x

[fsn32031-bib-0036] Shakeri, A. , D’Urso, G. , Taghizadeh, S. F. , Piacente, S. , Norouzi, S. , Soheili, V. , & Salarbashi, D. (2019). LC‐ESI/LTQOrbitrap/MS/MS and GC–MS profiling of *Stachys parviflora* L. and evaluation of its biological activities. Journal of Pharmaceutical and Biomedical Analysis, 168, 209–216. 30825804 10.1016/j.jpba.2019.02.018

[fsn32031-bib-0037] Shakeri, A. , Khakdan, F. , Soheili, V. , Sahebkar, A. , Rassam, G. , & Asili, J. (2014). Chemical composition, antibacterial activity, and cytotoxicity of essential oil from *Nepeta ucrainica* L. spp. kopetdaghensis. Industrial Crops and Products, 58, 315–321. 10.1016/j.indcrop.2014.04.009

[fsn32031-bib-0038] Shakeri, A. , Soheili, V. , Karimi, M. , Hosseininia, S. A. , & Fazly Bazzaz, B. S. (2018). Biological activities of three natural plant pigments and their health benefits. Journal of Food Measurement and Characterization, 12(1), 356–361. 10.1007/s11694-017-9647-6

[fsn32031-bib-0039] Singh, R. P. , Murthy, K. N. C. , & Jayaprakasha, G. K. (2002). Studies on the antioxidant activity of pomegranate peel and seed extracts using in vitro models. Journal of Agricultural and Food Chemistry, 50(1), 81–86. 11754547 10.1021/jf010865b

[fsn32031-bib-0040] Stratford, M. , & Eklund, T. (2003). Organic acids and esters. Food preservatives (pp. 48–84). Springer.

[fsn32031-bib-0041] Taechakulwanijya, N. , Weerapreeyakul, N. , Barusrux, S. , & Siriamornpun, S. (2016). Apoptosis‐inducing effects of jujube (Zǎo) seed extracts on human Jurkat leukemia T cells. Chinese Medicine, 11, 15 10.1186/s13020-016-0085-x 27042202 PMC4818408

[fsn32031-bib-0042] Tohidi, B. , Rahimmalek, M. , & Arzani, A. (2017). Essential oil composition, total phenolic, flavonoid contents, and antioxidant activity of *Thymus* species collected from different regions of Iran. Food Chemistry, 220, 153–161. 27855883 10.1016/j.foodchem.2016.09.203

[fsn32031-bib-0043] Tohma, H. , Gülçin, İ. , Bursal, E. , Gören, A. C. , Alwasel, S. H. , & Köksal, E. (2017). Antioxidant activity and phenolic compounds of ginger (*Zingiber officinale* Rosc.) determined by HPLC‐MS/MS. Journal of Food Measurement and Characterization, 11(2), 556–566. 10.1007/s11694-016-9423-z

[fsn32031-bib-0044] Vahedi, F. , Fathi Najafi, M. , & Bozari, K. (2008). Evaluation of inhibitory effect and apoptosis induction of *Zyzyphus Jujube* on tumor cell lines, an *in vitro* preliminary study. Cytotechnology, 56(2), 105–111. 10.1007/s10616-008-9131-6 19002848 PMC2259261

[fsn32031-bib-0045] Velasco, J. , Dobarganes, C. , Holgado, F. , & Márquez‐Ruiz, G. (2009). A follow‐up oxidation study in dried microencapsulated oils under the accelerated conditions of the Rancimat test. Food Research International, 42(1), 56–62.

[fsn32031-bib-0046] Vermerris, W. , & Nicholson, R. (2007). Phenolic compound biochemistry. Springer Science & Business Media.

[fsn32031-bib-0047] Wang, L. , & Weller, C. L. (2006). Recent advances in extraction of nutraceuticals from plants. Trends in Food Science & Technology, 17, 300–312. 10.1016/j.tifs.2005.12.004

[fsn32031-bib-0048] Wilfred, V. , & Nicholson, R. (2006). Phenolic compound biochemistry. Springer.

[fsn32031-bib-0049] Wojdyło, A. , Figiel, A. , Legua, P. , Lech, K. , Carbonell‐Barrachina, Á. A. , & Hernández, F. (2016). Chemical composition, antioxidant capacity, and sensory quality of dried jujube fruits as affected by cultivar and drying method. Food Chemistry, 207, 170–179. 10.1016/j.foodchem.2016.03.099 27080894

[fsn32031-bib-0050] Xu, B. , & Chang, S. (2007). A comparative study on phenolic profiles and antioxidant activities of legumes as affected by extraction solvents. Journal of Food Science, 72(2), S159–S166. 10.1111/j.1750-3841.2006.00260.x 17995858

[fsn32031-bib-0051] Xu, C. , Zhang, Y. , Cao, L. , & Lu, J. (2010). Phenolic compounds and antioxidant properties of different grape cultivars grown in China. Food Chemistry, 119(4), 1557–1565. 10.1016/j.foodchem.2009.09.042

[fsn32031-bib-0052] Yi, W. , Akoh, C. C. , Fischer, J. , & Krewer, G. (2006). Absorption of anthocyanins from blueberry extracts by caco‐2 human intestinal cell monolayers. Journal of Agricultural and Food Chemistry, 54(15), 5651–5658. 10.1021/jf0531959 16848559

[fsn32031-bib-0053] Zhang, H. , Jiang, L. , Ye, S. , Ye, Y. , & Ren, F. (2010). Systematic evaluation of antioxidant capacities of the ethanolic extract of different tissues of jujube (*Ziziphus jujuba* Mill.) from China. Food and Chemical Toxicology, 48(6), 1461–1465. 10.1016/j.fct.2010.03.011 20230870

[fsn32031-bib-0054] Zhen, J. , Villani, T. S. , Guo, Y. , Qi, Y. , Chin, K. , Pan, M.‐H. , Ho, C.‐T. , Simon, J. E. , & Wu, Q. (2016). Phytochemistry, antioxidant capacity, total phenolic content and anti‐inflammatory activity of *Hibiscus sabdariffa* leaves. Food Chemistry, 190, 673–680. 10.1016/j.foodchem.2015.06.006 26213025

